# The New York Institute of Stomatology

**Published:** 1904-08

**Authors:** 

**Affiliations:** The New York Institute of Stomatology


### Reports of Society Meetings.

### THE NEW YORK INSTITUTE OF STOMATOLOGY.

A REGULAR meeting of the Institute was held at "The Chelsea," No. 222 West Twenty-third Street, New York, on Tuesday evening, April 5, 1904, the President, Dr. Brockway, in the chair.

There being no communications on theory and practice, the paper of the evening, "A Plea for Thorough Dental Education," was read by Dr. Leo Green.

(For Dr. Green's paper, see page 585.)

*Dr. Eugene H. Smith.*—I am in full sympathy with that part of Dr. Green's paper relating to more thorough preliminary training. I would like first, however, to say a few words relative to his criticism of the conduct of the dental schools. The errors he points out are in the main true, but there is a good reason for these errors. First, as to the repetition of lectures. I find this repetition is necessary with the present class of students. I have had a chance to study this question thoroughly, having had to deal with students presenting widely differing degrees of preliminary education, from the minimum requirement to the man who has his college degree. I have asked these different men as to the wisdom of repeating lectures. In every instance the college man has replied it was unnecessary,—that he did not need to have the same thing told him twice. The poorly trained man invariably says, "I was unable to properly grasp the subject at first." I think, however, that there are very few colleges to-day that repeat lectures three years in succession. Most colleges repeat the junior and senior lectures. The correction of all this is a higher preliminary education.

The essayist speaks of the substituting of apprenticeship in an office for college work. This does not exist in any of our colleges, so far as I am aware; certainly not in Harvard. He also speaks of annual examinations, and the large number of failures in the last year as compared with other years. I can only say that this is not so in my experience. We generally register a class of fifty members. The first year is their Waterloo, because they are taking anatomy, physiology, histology, bacteriology, and physiological chemistry along with the medical students in the Harvard Medical School. Of this number as many as thirty per cent. fail in a majority of their examinations. If a student successfully passes his first year he is a very good man.

Another criticism was that of employing young teachers. I think every dean who has the selecting of instructors will bear me out in saying that the average graduate of twenty years ago is not a fit man for an instructor in the present dental school. While it is desirable that we should have men more experienced in practice, still such a man is apt to have many hobbies. He is set in his methods, and is apt to be a dangerous man for the young student to meet, but a most valuable man after the student has become more matured. Then again it is difficult to get the older men as teachers. They are busy men, and every hour they teach is a financial loss to them, more so in dentistry than in medicine. The dental man who gives his time to charity or to teaching does it at a greater cost to himself than does the medical man.

The essayist says that the college authorities should make it plain to the impossible student, at the earliest possible moment, that his sphere of usefulness is elsewhere. I think the majority of men who have charge of the education of students do this very thing. It is a difficult matter, however, to determine. Many men who do discouraging work to begin with turn out to be first-class men. So a teacher takes a great deal upon himself who says to a young man, "You have missed your calling; your field of usefulness is elsewhere." Again, the student may think otherwise.

The essayist speaks of the habit of practitioners concealing useful ideas. The men with whom I have had the honor to be associated have been perfectly free with their ideas, and their laboratories and operating-rooms have not been locked to their fellow-workers.

A proper preliminary education is essential to any professional education. There certainly can be no question about that. The only difference of opinion that can exist is as to what that preliminary education shall be. Some go so far as to say that the practitioner in dentistry should receive the same education as the specialties in medicine; that the dental practitioner should have a medical degree. There is strong logic in their arguments, and it may come to this. On the other hand, we will find other men, like our essayist, who will argue for a college training. I feel that I can enter upon the discussion of this subject without prejudice, as I hold but the one degree, that of dentistry. As far as my experience as a teacher goes, I have invariably found that the man with the right kind of a college training makes the best dental student, and can do more work in a given time than the man can whose preliminary training is represented by two years' work in a high school. This being true, it follows that with high preliminary requirements, and a college course of three years of nine months each, much more can be done in the way of advancing our professional standing than can be accomplished in a four years' course with immature and improperly trained men.

*Dr. B. Holly Smith.*—There are some misstatements in the paper as to the actual facts, unless the essayist has inside information of which I am not possessed. I have been a teacher for twenty-one years and am a member of the Association of Dental Faculties, and I stand here to-day to say that the system of dental education in this country is not faultless, but it is in every way far above that described by your essayist. I know of no lax treatment of foreign students. Do not we have associates appointed in all the countries of Europe, and are not we more jealous of the good behavior of men who go abroad than of any other class of students? So it seems unfair that we receive this statement as an absolute fact.

As to our preliminary training. Why does not this organization appoint a committee to formulate a proper plan? Do we not resolve too much? Do we not discuss too much? Let us *do* something. Every one of us knows that the dental student should have a special preliminary training. What we need to determine is what that special training ought to be. It would seem desirable that instead of the language courses now necessary for college entrance, a course in manual training be substituted for those students contemplating entering the profession of dentistry. It seems to me here in your Columbia University such a course might be given, not unlike the course for students in medicine. A kind of manual-chemical-physical course. We do want more finger-work. We want training along certain special lines. Now, instead of discrediting those who hold for a four years' course, why not allow anybody who has secured an A.B. degree in the chemical-biological course a credit of one year of that four years' course? The proposition of the essayist as to a nine months' school year does not at all meet with my approval. I would say broadly that there are two distinct propositions made in the paper that I should oppose. First, I do not favor an A.B. degree, excepting one along the lines of the student's life work. Otherwise it is wasted time. Secondly, I am opposed to a nine months' course. I grant that in Boston, where pupils are not subjected to any great changes of temperature, they can manage to work for nine months. But this cannot be done in the Southern States. Take Atlanta, Louisville, or Nashville. It is simply impracticable. Then again, many of our dental students are self-supporting, and it is necessary for them to have the interval between sessions to secure the wherewithal for a return to college. I do not at all agree with the essayist when he says that a greater part of our dental students are men who have tried mercantile pursuits and failed. It is not so in Baltimore, and I do not believe it is so anywhere. A large percentage are men who are much more capable than the average salesman or clerk. I have never heard of any credit being given a student for laboratory or office work. Certainly it is against the rules, and I rather think the essayist is mistaken. Dr. Smith says that the time is not yet ripe for an A.B. degree. It never will be ripe for such an A.B. degree as many of the colleges are granting. Is it not some evidence of education to have secured the degree of D.D.S.? Is it not necessary that a man should study, should read, should think, should experiment; that he should use his hands? Certainly this all-around training makes him a larger man than would development of the mental faculties alone. I have known college graduates who could not take the bridle from a horse, who did not know how to open a gate. The dentist is to-day in my judgment the most resourceful man in the world. So far as recognition is concerned, it bothers me not at all. It depends upon the individual whether he is recognized or not.

*Dr. Edward C. Kirk.*—I wish to speak about some things that have been touched upon and also about some things that have not. I know very little about the majority of dental schools, but I am surprised to learn what I have to-night,—that they are in such a state of degeneracy. I was very much interested in the suggestion for a definition of what constitutes a high educational standard. I have written something about that and said something about it, and I would like to get in the minds of my professional colleagues this idea that there is a difference and a great big one between the quality and the quantity of an educational standard, and its adaptability to a given purpose. I quite agree with Dr. Holly Smith when he says that any old A.B. degree will not do. It must represent a certain kind of training adaptable to the purpose of preparing a man for entering upon the study of dentistry. The question of a Latin requirement has been touched upon. I hold that a year or two years of Latin is insufficient except for the discipline which any study affords. Other than the discipline common to all education, Latin is valuable for two purposes, either for the study of Latin literature or as a side-light upon the study of English. On the theory of Goethe, that he who does not know a foreign language does not know his own, how much does a student get out of one or two years of Latin? Of how much value is it as a preliminary training for admission to the professional study of dentistry? I wish to quote some extracts from a correspondence with a well-known gentleman regarding the uses and purposes of Latin as taught in our schools. "So far as I have ever seen, the training of Latin in the preparatory schools is as bad as it can be. It was bad in Milton's time, and the faults that existed then seem to exist now." I was present at a commencement when the president said, "Qui gradum doctores dentarii accedunt," but not one of the students knew enough of Latin to go up and get his diploma. That might have happened in any dental school under the present entrance standards. If a man does not know enough Latin to come up and get his diploma when he is asked to do so in Latin, of what use is it to him. In order to get the use of Latin a student should have a dozen years of it; he would then be in a position to read Latin literature and would understand his English better. I heartily agree with Dr. Smith, of Harvard, that the man with the college training is the better man all round, and personally I am sorry that the opportunity was not given to Harvard University to try this experiment which they are determined to try independently of the Association of Faculties. The objection that I have to the position as expressed by Dr. Smith is that this is the ideal which he thinks ought to be attained by all colleges. I want to have this recognized, that we are not making soldiers. We have no such thing as a system of dental education. We have, instead, systems of dental education. We are not duplicating and multiplying men of the same caliber or capacity. Some must become strong in certain ways, and they will necessarily be weak in others. I believe there is room to-day for an institution that will try just this experiment that Harvard University means to try, but on that account it should not be said that as it is good for a certain number, therefore everybody must do the same thing. This special training will be manufactured into that thing called culture, and there is undoubtedly room for a great body of such cultivated men. But do you suppose they are the best men to serve the public everywhere? I do not think so. Such a man will naturally gravitate to the cities where culture abounds, and where he will be in sympathetic touch with those of his own class, and he would be unhappy in any other environment. From this class of men will doubtless be produced investigators and teachers, and of both these we need many. But there is room for a much larger number of men who will be empirics. The average medical practitioner, when asked why he treats a case thus and so, will give you an answer based on the rule of thumb. There is room in my judgment for that larger class of men who have only the preliminary education furnished by the State to practise among that class of people commonly spoken of as the "common" people. A man who is in sympathy with them. Then again there is a great class of men who are unable, because they are brought face to face with the bread-and-butter problem, to go into the field of education beyond that which the State furnishes. Indeed, the high school furnishes a very good training to-day. Who made dentistry; was it college graduates? I think not. Or if some of our dental pioneers were college men, is it not true that the average A.B. of thirty-five or forty years ago is no better educated than the high-school graduate of to-day? So I think it is a wrong method to make an A.B. degree a necessary qualification for entrance into the dental profession.

The essayist, with a wave of his hand, dismisses the whole subject of State sovereignty, saying that this difficulty about interchange of licenses could be overcome by the creation of a national standard. Certainly it could, but that involves an amendment to the Constitution of the United States, and that is a question affecting State sovereignty, which is a very difficult problem. The last time that State sovereignty was practically considered in this country was in the early youth of most of us, when several hundreds of thousands of able-bodied men laid down their lives in the discussion of it. I do not believe there can be any such amendment making an obligatory national standard possible, however desirable it might be.

As to the question of commercialism and what the essayist has designated as English law, I doubt if it is English law. What he has quoted is merely a contract. It might hold under English law, but I doubt if it would hold here.

*Dr. Faneuil D. Weisse.*—I am an optimist in the full sense of the word and especially so with regard to what the dental institutions of this country have accomplished in establishing dentistry as a profession. I look upon all plans as regards preliminary education as unnecessary, as it has had and is going through its natural evolution, in spite of sporadic spurts by individual institutions. In New York State preliminary educational requirements to enter the profession have been steadily progressive since 1894, and every step forward has been conservative and permanent, with the exception of the experience of 1897, when it was realized that the advance to a 48-count requirement for dentistry was premature.

The way of the dental institutions of the United States has been hard, indeed, as they never have received from contributors to the dental journals any encouragement or commendation for their work. On the contrary, the dental journals of the country give us periodical freshets of articles criticising straw-like shortcomings in the general work.

Turning from the minor details of college work criticised by the author of the paper, let us realize that the present status of dentistry as a profession is due to the aggregate results of the work of our dental institutions.

The calling of the practice of dentistry has been a profession little more than sixty years; before that it was only a handicraft. *No calling is a profession until institutions have been established with a defined scientific curriculum for education and training for its practice.* Surgery was at one time a handicraft, and the surgeon was the servant of the physician and only became his peer after medical institutions had made provision in the medical curriculum for his education as a surgeon.

The birth of the calling of dentistry as a profession was at the sending forth, with the degree of D.D.S., of the first class of the Baltimore College of Dental Surgery, in 1841. Since that time the other dental institutions of the country have been established to educate for a profession.

Some critics have held that the creation of the degree of D.D.S. was a mistake; that all practitioners of dental surgery should be practising under the degree of M.D. To my mind the phenomenal growth of the dental profession is due to the fact that the degree of D.D.S. was created, and that a scientific curriculum for education to enter its ranks was evolved independently of the M.D. degree, thereby enabling the dental student to concentrate his energies within definite lines of his special education unincumbered by subjects which have no bearing on his specialty of general medicine.

Under these advantageous conditions the dental institutions of the United States have made the D.D.S. degree the evidence of special professional education and skill throughout the world where dental surgery is practised.

To-day the D.D.S. degree has won its peerage of the M.D. degree, and that from the hands of the mother profession, in that the American Medical Association has recently voted the D.D.S. degree to membership.

To-day European and Pan-American institutions are organizing along the lines of those of the United States. Only the other day I received a letter from a member of the Parliament of New Zealand asking for information as to the organization of the New York College of Dentistry, in view of the establishment of a dental department of the University of New Zealand.

With the above work achieved by our dental institutions before them, the dental profession of the United States have reason to be proud of the results accomplished by their professional institutions during the short life history of their profession—as such—of only sixty years. When the history of the nineteenth century is written it will record the fact that in the United States, during the last six decades of the century, the handicraft of dentistry rose to the dignity of a profession, by the establishment of special institutions with a specialized scientific curriculum for its education and training, and under the *ægis* of the D.D.S degree.

*Dr. Hopkins.*—I certainly am very grateful to the society for bringing this subject up for discussion, and while the essayist has come in for a great deal of criticism, I think he may console himself in the thought that he is a martyr to the cause of advanced dental education, and may congratulate himself that he has created a greater interest in the subject than has ever been expressed before at any dental meeting. I am delighted that this dual experiment of a three and a four years' course is going to be tried, and I am not at all sure what the result is going to be. I am not sure but that men equipped as will be the men who enter the Harvard Dental School will be able to accomplish as much or more in their three years than can the ordinary students in four. However, I do not agree with the essayist that any A.B. degree would be a suitable preliminary training. I think that anything which retards the taking up of the technical part of dental work retards the practitioner in the acquirement of manipulative skill. It may have been a faulty observation, but it has been my experience that men who have obtained their academic degree from institutions where they have received no manipulative training, do not acquire skill in practice as early nor as surely as those students who begin their dental work when they are four years younger. It is a question if the academic degree will be altogether helpful until it is changed so that technical requirements may be substituted bearing upon the professional training which is to follow.

Aside from this, I think most of the improvement in preliminary examinations should be upon the subject of English. I believe I can tell, after a student has written an essay of five hundred words, and after I have had fifteen minutes' conversation with him, whether that student is going to be able to understand the lectures in his course and the subjects in his text-books. I think the greatest failing in preliminary education to-day is in English. If a man, no matter whether he comes from the high school or from college, has a correct knowledge of English, it indicates a capacity to understand the theoretical work of his course in dentistry, and is a better preparation than any amount of Latin. There is great encouragement to the profession in the fact that so many representative men are interested in this question of higher dental education, and that each one is seeking for the greatest good of the profession, although perhaps there is lack of agreement as to how it can best be accomplished.

*Dr. E. A. Bogue.*—It seems to me that the statement has not yet been made as to what the dentist's education should be, though

I have heard some good English, some bad English, and precious little Latin. A good many years ago there were two or three medical men who took up the study of the mouth and its associated parts.

I do not think that a committee from this body could formulate a practical plan for dental education, nor any other committee. The ordinary dentist's work is to prevent pain or loss, or to replace lost parts. The craft began with the mechanical side. It has been enlarged until the medical branch includes prophylaxis. It has become, in the hands of the colleges and practitioners, a liberal profession. As a mechanical art there was very little to study, but as a liberal profession it has opened up the same field for study and observation as the other professions. The question now comes up as to whether Dr. Eggleston was right when he said that the best way of instructing a man in doing anything that his hands could do was to set him doing it. Dr. Holly Smith tells us that the climate of the country is so varying that we must have varying systems. Dr. Eugene Smith tells us that in certain States technical education is compulsory. We may infer that such States furnish the best preliminary education for dental students. Anyway, we shall undoubtedly find that when the dental instructors of this country shall have perfected their organization, and when they decide what they consider for the best for the students, they will adopt it, and that in the end we will come to a system applicable to the students of the entire country and the teaching will be such as will result in the giving to the profession such a body of men as will administer to the needs of their fellows in the most successful and advantageous manner. I have no doubt that hints from any of us will be thankfully received.

*Dr. Ottolengui.*—I have been delighted to hear with what perfect frankness this subject has been discussed this evening. The points seem to have all been covered by the gentlemen connected with the colleges. While I am a dentist, I have devoted a number of years to the study of entomology, and consequently the story of the "humbug" was familiar to me. I am going to risk another. A professor once asked one of the boys in his class to tell him what a moth is. The boy told him that a moth is an insect with a head, a body, from one to four eyes, one or two antennæ, and from one to four feet, with a pin through the middle of it. The professor said, "Where did you get that idea of an insect?" "From a study of the college collection," was the reply. The essayist's impression of the colleges of the country has been acquired more from similar papers and criticisms than from a study of the colleges themselves. As I have gone about the various cities I have made it my business, wherever there was a college, to visit that college, and I have, without exception, been impressed with the fact that, whether the college was a large college or a small college, the evident intent to do the best that could be done was conspicuously present in all of the schools. I entirely agree with what Dr. Weisse has said. It is well for us to bear in mind that whatever we are, we are the product of our educational institutions, and instead of berating them we should be proud of them and praise them. So far as I myself am concerned, my sympathies are entirely with the schools and what they are doing.

Dr. Kirk brought out the idea that one particular kind of dentist will not suffice for this broad American continent. There is no question but that the system to be adopted by Harvard will be eminently successful and will produce men of the highest attainments, but another class of dentists also is required,—dentists who will be willing to work for the lowly and for lowly fees. We have here in New York a very cosmopolitan centre, and we need men here not only of the highest culture, but also men who will be willing to work among the lower classes and for small stipends. For Thursday night I have accepted an invitation to address a society down in the Bowery, known as the Eastern Dental Society of New York. What litttle I know about these men has interested me. They are practising among an ignorant and cosmopolitan people who understand very little or nothing of the English language. These men are evidently not graduates of Harvard, but are not they doing a good work in this world, and was it not worth while that they were graduated from some school?

So I want to say, with the kindliest spirit in the world to the essayist, that this subject is much larger than his paper has covered or could cover. At the same time, it is in the hands of the very best men capable of solving it, the men who have charge of the colleges. Suppose the schools were all closed. Do you think the men who are criticising them could do better?

*Dr. Green.*—I feel very much like Daniel in the lions' den, for more than one reason; in the first place, because I do not know whether I am alive or not, and in the second place, because I am about as well able to defend myself as Daniel was. There are just one or two points that I wish to go over. The gentlemen who have discussed my paper to-night represent, with one exception, the side of the Associated Faculties, and necessarily their discussion is one-sided. I want to quote once more from Dr. Carlton's paper, not only because it is apropos, but also because, contrasted with the remarks of some of the previous speakers, it indicates that even the Associated Faculties (to whom Dr. Holly Smith so frequently referred) are not entirely agreed in their "ideas" for dental education.

"Higher education is not a question alone of preparing great men for great things; it must also prepare little men for greater things than they would have otherwise found possible. As a good beefsteak disappears in a man and reappears as energy and vigor, so is education absorbed, and reappears as mature judgment, mental balance, keen, acute, grasping intellect. Is there a practitioner among us not holding the degree of Bachelor of Science, Bachelor of Arts, Doctor of Medicine, or otherwise, who does not bitterly regret opportunity slighted or unavailable? He would indeed be glad to possess them, not for the mere degree's sake alone, but for the education's sake. . . . I trust I have said enough to prove to you that the opportunities of modern dentistry are so new and vast that an ampler education is needed by the practitioner of the future. We cannot give them that education in a four years' course at a Dental College. Let him take ten, if necessary, with the preliminary; it will be worth the while, and his place in the professional world will wait for him. The four-year courses in our dental schools will be well and completely filled with good instruction and practical work."

Now, gentlemen, my motives in presenting this paper have been entirely misunderstood. I did not come here to try to teach the men who are in charge of the various dental colleges of this country how to run their institutions; but, on the other hand, I do not agree with Dr. Ottolengui that we must leave the faults where we find them. I am perfectly willing to take all the fair criticism I have received to-night, if I have provoked an honest discussion of this subject. I think Dr. Kirk was disposed to jest with my paper. Some of the indictments he brings against dental education in his various articles are almost as severe, if not more so, than mine. In his article in the January, 1904, *Dental Cosmos,*

on the "Scientific Method in Dentistry," he deplores the empirical methods in our instruction, etc., and regrets the "attitude of downright contempt" towards scientific methods. This is somewhat at variance with his attitude and remarks this evening. I quote from his article as follows:

"There seems to be a widespread feeling that the scientific method in dentistry is a sort of mental jugglery by which certain men find diversion in mystifying others who have no task for that kind of entertainment. I do not think I have overdrawn the situation, for the belief seems to be quite general that there are two aspects to dentistry,—one denominated 'theory' and the other denominated 'practical.' It is voiced generally by State boards of dental examiners; it is the current belief of dental students. Dental college faculties are by no means blameless in this connection. . . . We must understand always that the school is its corps of teachers, and that everything else in the institution is merely accessory thereto. No man should be permitted to teach, to form the habits of mind, the ideals, of the recruits of our profession who has not in some way obtained that breadth of training which means not only knowledge of the data of science, but scientific culture as well. It is not what he teaches so much as how he teaches that indicates his fitness and determines the type of practitioner he creates. In proportion as the teacher uses the scientific method does he eradicate from the minds of his students the empirical method and improve their mode of practice by putting it upon a rational basis."

Empirical methods, as I understand them, are those which depend upon isolated experiments and observation without due regard to scientific principles. I find our educational methods empirical in that they neglect the first principle of scientific procedure,—a lack of preliminary training.

Most of the criticism here to-night has been directed against my suggestion for a baccalaureate degree as a preliminary requirement. All who have discussed the paper have either ignored or forgotten the fact that I distinctly stated what I considered to be a proper baccalaureate degree, and not "any old degree," as stated by Dr. Holly Smith. I am afraid that Dr. Kirk referred to an amendment of the constitution in a spirit of ridicule. I think it would be possible for an understanding to exist among the organized boards of examiners, whereby an interchange of licenses might be effected without a national calamity.

Dr. Holly Smith has mentioned the fact that the climatic conditions in many States makes a nine months' course impracticable. I would like to ask the doctor if in these same States the climatic conditions prevent a man from working more than nine months of the year.

A great many misstatements are charged against this paper. The only reason that I did not mention specific incidents was, that I desired to avoid all personalities. I regret that some of the men who discussed my paper should have accused me of misstatements. To any of these men I shall be very glad indeed to give specific instances in proof of what I have stated.

I am very grateful to you, gentlemen, for having given me so much of your attention.

Adjourned.

FRED. L. BOGUE, M.D., D.D.S.,
*Editor The New York Institute of Stomatology.*

